# Clinicopathological features and risk factors for local recurrence of breast phyllodes tumors: a dual-center, 12-year, retrospective study

**DOI:** 10.3389/fonc.2025.1538523

**Published:** 2025-05-26

**Authors:** Yiwen Li, Heyan Chen, Fengjiang Qu, Xiaoying Qu, Qihe Zhang, Kexuan Feng, Xiaoduo Li, Huimin Zhang, Zhimin Fan

**Affiliations:** ^1^ Department of Breast Surgery, The First Hospital of Jilin University, Changchun, Jilin, China; ^2^ Department of Breast Cancer, The First Affiliated Hospital of Xi’an Jiaotong University, Xi’an, Shaanxi, China; ^3^ Department of Emergency Surgery, The First Hospital of Jilin University, Changchun, Jilin, China; ^4^ Clinical Medicine, Xi’an Jiaotong University Health Science Center, Xi’an, Shaanxi, China

**Keywords:** breast phyllodes tumor, fibroepithelial tumor, rare breast tumor, breast surgery, local recurrence (LR)

## Abstract

**Purpose:**

To clarify the risk factors of phyllodes tumor (PT) for local recurrence (LR).

**Methods:**

Data from 829 patients with pathologically confirmed benign, borderline and malignant PT of the breast, diagnosed from between 2011 to 2023, were retrieved from the electronic databases of the First Hospital of Jilin University and the First Affiliated Hospital of Xi’an Jiaotong University. Kaplan-Meier curves and Cox proportional-hazards model were conducted to determine the independent risk factors for LR in each group.

**Results:**

Of 829 PT patients, 634 (76.5%), 142 (17.1%), and 53 (6.4%) were diagnosed with benign, borderline, and malignant PT, respectively. The LR rates were 5.4%, 9.9%, 13.2%, respectively. The median patient age was 38 years and the median follow-up time was 2.8 (range, 0.2-12.1 years). Of these patients, 13 (2.1%) were diagnosed with benign bilateral PT. Multivariate analysis identified bilateral involvement as a risk factor for LR of benign PT (*p*=0.010). Also, univariate analysis identified young age (≤35 years, *p*=0.046) as an independent risk factors for LR of borderline PT. Of the patients with malignant PT, univariate analysis found that breast-conserving surgery (BCS) (*p*=0.008) were associated with an increased risk for LR of malignant PT.

**Conclusions:**

Bilateral PT was a risk factor for LR of benign PT, young age (≤35 years) was associated with poor prognosis of borderline PT, BCS were high risk factors for LR of malignant PT. This study identifies LR risk factors based on tumor grading, which contributes to individualized clinical risk assessment. Future research could further explore how to incorporate these factors into clinical decision-making models for PT and other soft tissue tumors.

## 1 Introduction

Phyllodes tumor (PT) of the breast is a rare and complex growth, accounting for 2%–3% of all fibroepithelial neoplasms and 0.3%–1.0% of all breast tumors (1). PT most commonly occurs in women aged 45–49 years, while comparatively infrequent in men (2). The World Health Organization classifies PT into three categories based on histological features: benign, borderline and malignant (as shown in **Table 1**).

**Table 1:** Histological characteristics of benign, borderline, and malignant PT (3).

Feature	Benign	Borderline	Malignant
cytologic atypia	mild or no	mild to moderate	marked increase
tumor border,	well-defined	focally permeative	permeative
stromal cellularity	Mild	moderate	marked
stromal overgrowth	Absent	absent or focal	marked and diffuse
mitosis	0–4/10 HPF	5–9/10 HPF	≥10/10 HPF

HPF, High Power Field.

Of all breast phyllodes tumors, the prevalence of benign PT is notably higher than malignant PT (60% vs. 25%–30%, respectively) (4, 5).

Clinical presentation in many phyllodes tumor is a palpable breast mass, but benign PT is ofen misdiagnosed as fibroadenoma. Meanwhile, differentiation of borderline and malignant PTs from breast carcinoma can be challenging. An accurate diagnosis relies on pathological examination following complete tumor excision. However, there is currently no consensus regarding the most appropriate surgical type.

PT have a high risk of recurrence, but the factors affecting their recurrence remain unclear. Therefore, the aim of this study was to clarify the clinical and pathological characteristics in addition to risk factors associated with LR of PT to provide evidence-based guidance for clinical diagnosis and treatment.

## 2 Materials and methods

### 2.1 Study approval and patient consent

The study protocol was approved by the Ethics Committees of the First Hospital of Jilin University(approval no. 2024–627) and the First Affiliated Hospital of Xi’an Jiaotong University (approval no. XJTU1AF2021LSK-238) and conducted in accordance with the ethical principles for medical research involving human subjects described in the Declaration of Helsinki.

### 2.2 Patients and variables

**Figure 1:**
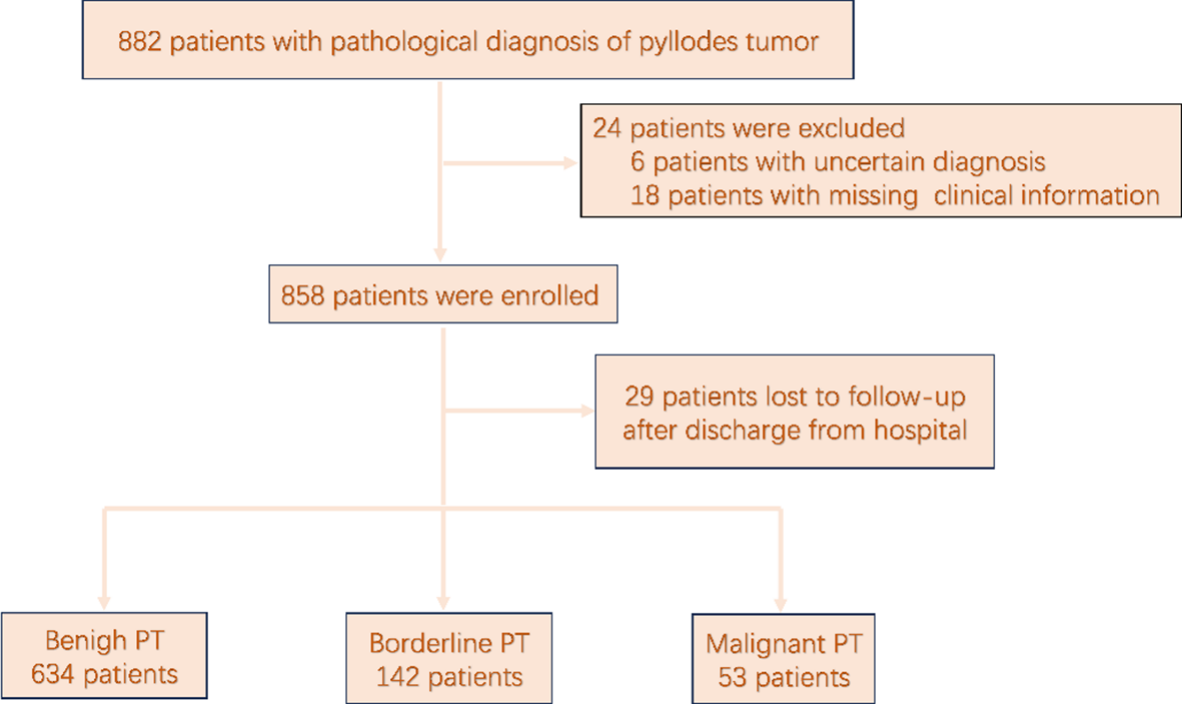
Study flowchart.

The medical records of 882 patients with pathologically confirmed PT following resection from August 2011 to October 2023 were retrieved from the electronic databases of the Breast Surgery Departments of the First Hospital of Jilin University and the First Affiliated Hospital of Xi’an Jiaotong University. A flowchart of the patient selection process in presented in **Figure 1**. Of the 882 patients with pathologically confirmed PT, 829 (94.0%) who had undergone surgical resection and had a pathologically confirmed diagnosis of PT, with complete follow-up data available met the inclusion criteria and were included for analysis. In accordance with the classification criteria proposed by the World Health Organization, PT was classified as benign, borderline, or malignant.

The patient baseline characteristics included age at initial diagnosis, body mass index (BMI), tumor size, laterality, estimated blood flow by ultrasonography, and type of surgery.

Groups were established based on age at initial diagnosis (≤ 35vs.>35 years) (6–8) and tumor size (≤ 50vs.> 50 mm) (2). BMI was calculated as weight (kg)/height (m)². Based on the calculated BMI, patients were classified as overweight (BMI>24) or normal weight (BMI≤ 24) (9). Vacuum-assisted excision (VAE) is a minimally invasive therapeutic technique derived from vacuum-assisted biopsy (VAB). As the technology has advanced, VAE has seen increasingly widespread use in treating benign breast lesions (10). Therefore, in our study, the surgical approaches included vacuum-assisted breast excision (VAE), breast-conserving surgery (BCS), and total mastectomy (TM).

The outcome variable was LR, defined as cytologically or histologically confirmed LR of PT involving the ipsilateral breast or chest wall (including the ipsilateral axilla, internal breast, subclavian or supraclavian lymph nodes) at ≥60 days after initial surgery. Local recurrence-free survival (LRFS) was defined as the survival time from the date of surgery to the first determination of LR. Distant metastasis (DM) is defined as the spread of neoplastic components to distant organs. For all patients with confirmed LR of malignant PT, the pathological records were scrutinized to ascertain disease status.

### 2.3 Statistical analysis

All statistical analyses were conducted using IBM SPSS Statistics for Windows, version 26.0. (IBM Corporation, Armonk, NY, USA and Prism 10.0 software (GraphPad Software, Inc., San Diego, CA, USA). Descriptive analysis of the three patient groups included categorical data (frequency and percentage) and continuous data (median, interquartile range [IQR]). The Kaplan-Meier method was used to estimate the 5-year LRFS, and the log-rank test was performed to compare differences between groups.

Univariate analysis with the Cox proportional-hazards model was conducted to prevent the omission of variables with statistical significance for LR due to confounding factors. Variables with a *p*-value<0.1 *(* 11)were included in the multivariable Cox regression analysis to identify independent risk factors for LR in each patient group. A two-sided *p*-value < 0.05 was considered statistically significant.

### 2.4 Follow-up

Patients diagnosed with PT were recommended to undergo breast ultrasonography every six months after surgery. For those with borderline or malignant PT, annual mammography and systemic examinations were additionally advised. Patients were followed up by telephone or the outpatient electronic medical record system. Baseline characteristics, imaging results, surgical records, postoperative pathological results, and postoperative recurrence were recorded. Based on the longest observed time to LR, the maximum follow-up period was 12.1 years after initial surgery.

## 3 Results

### 3.1 Baseline characteristics

**Figure 2:**
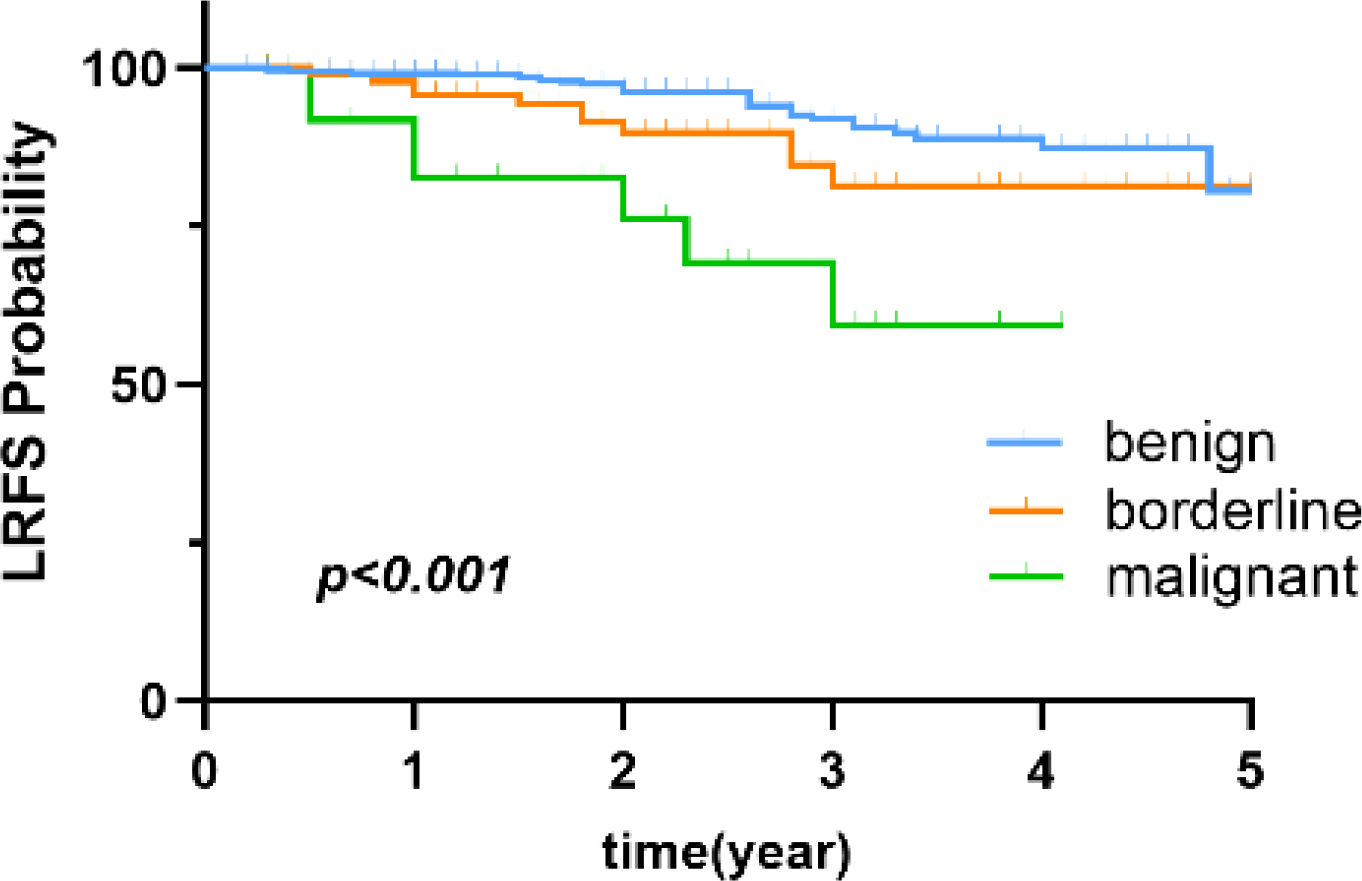
Comparison of 5-Year LRFS for benign, borderline, and malignant PT.

The clinicopathological characteristics of the 829 patients are summarized in **Table 2**. All 829 patients were female. Of these patients, 634 (76.5%), 142 (17.1%), and 53 (6.4%) were diagnosed with benign, borderline, and malignant PT, respectively. The median follow-up time was 2.8 (range, 0.2-12.1) years. Comparisons of the 5-year LRFS for benign, borderline, and malignant PT are shown in **Figure 2**.

**Table 2:** Patient, tumor, and treatment characteristics (N = 829).

Characteristics		Benign PT (n=634)	Borderline PT (n=142)	Malignant PT (n=53)
		n (%)	n (%)	n (%)
**Age(y)**	≤35	315 (49.7)	31(21.8)	10(18.9)
	>35	319 (50.3)	111(78.2)	43(81.1)
**BMI(kg/m2)**	≤24	460(72.6)	96(67.6)	35(66.0)
	>24	174(27.4)	46(32.4)	18(34.0)
**Tumor size(mm)**	≤50	547(86.3)	82(57.7)	28(52.8)
	>50	87(13.7)	60(42.3)	25(47.2)
**Laterality**	Left	313(49.4)	83(58.5)	21(39.6)
	Right	308(48.5)	59(41.5)	32(60.4)
	Bilateral	13(2.1)	0	0
**Ultrasound** **blood flow signals**	Yes	197(31.1)	82(57.7)	31(58.5)
	No	446(68.9)	60(42.3)	22(41.5)
**Surgery type**	BCS	451(71.1)	110(77.5)	18(34.0)
	TM	5(0.8)	25(17.6)	35(66.0)
	VAE	178(28.1)	7(4.9)	0
**LR**	Yes	34(5.4)	14(9.9)	7(13.2)
	No	600(94.6)	128(90.1)	46(86.8)
**DM**	Yes	0	0	7(13.2)
	No	634(100)	142(100)	46(86.8)

LR, local recurrence; DM, Distant metastasis; BCS, breast-conserving surgery; TM, total mastectomy; VAE, vacuum-assisted breast excision.

### 3.2 Benign PT

For patients with benign PT, the median time to LR was 2.6 (IQR, 1.7-3.3) years, with the majority occurring within 5 years of initial diagnosis, the 5-year local recurrence rate (LRR) was 4.7%, the LRR of benign PT was 5.4%. the median age at initial diagnosis was 36 (IQR, 26-44) years, and the median tumor size was 27.6 (IQR, 20.0–39.8) mm.

**Figure 3:**
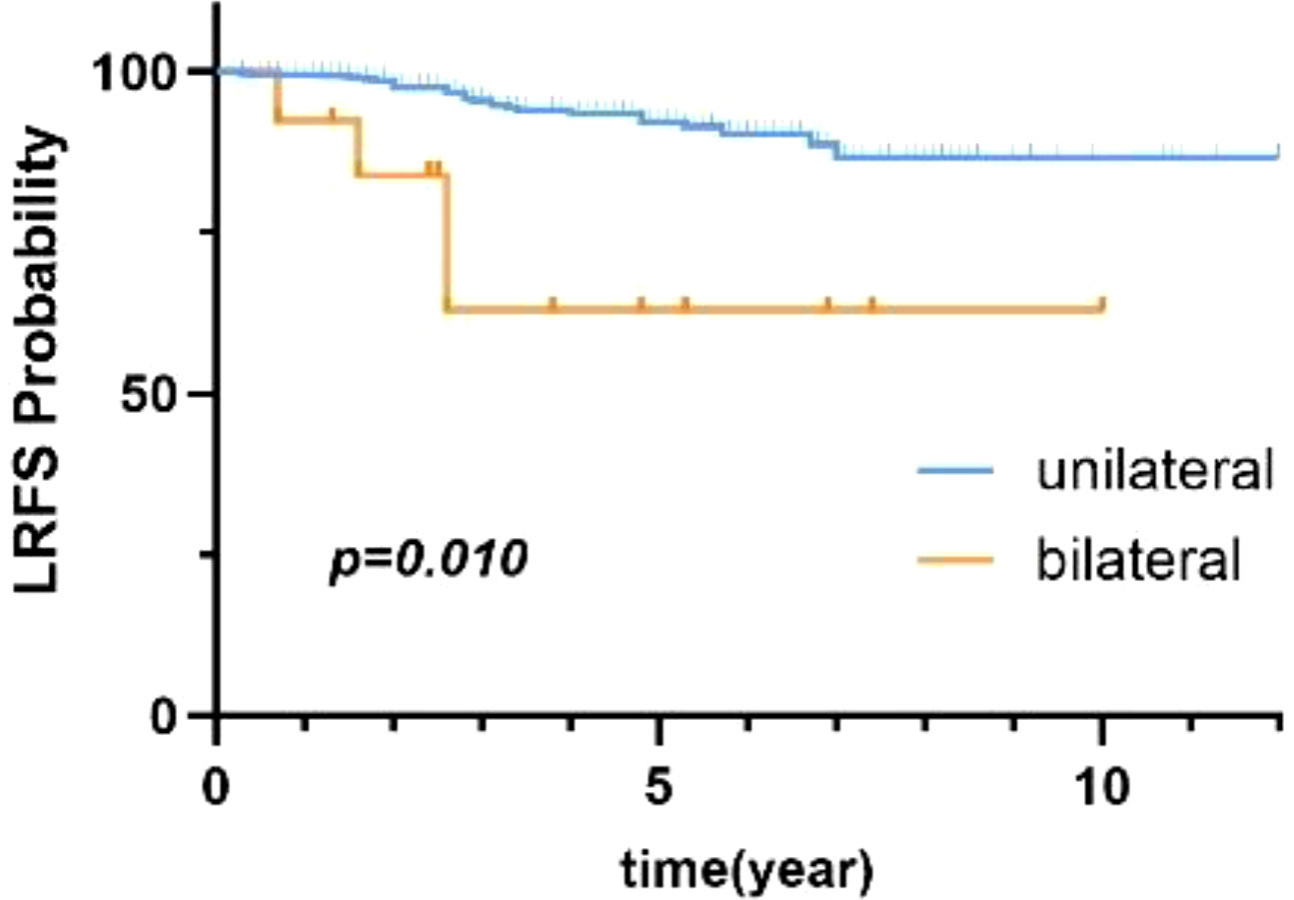
LRFS of benign PT based on laterality.

Univariate analysis with the Cox proportional-hazards model revealed that age, BMI, tumor size, and laterality were prognostic factors for patients with benign PT (*p* < 0.1). Thus, these four factors were included in multivariate analysis. As shown in **Table 3**, laterality was the only factor influencing local control in patients with benign PT. Additionally, compared to unilateral PT, bilateral breast PT significantly increased the LRR of PT (HR=3.99, 95% CI:1.38-11.48),. There was a statistically significant difference in 5-year LRFS between patients with unilateral and bilateral PT (*p*=0.010) (**Figure 3**).

**Table 3:** Univariate and multivariate Cox regression models for variables associated with benign PT.

Characteristics		Univariate analysis		Multivariate analysis	
		Hazard ratio (95% CI)	*p*-value	Hazard ratio (95% CI)	*p*-value
**Age (years)**	≤35 (Ref)				
	>35	0.51(0.25-1.02)	***0.058***	0.67(0.32-1.38)	0.270
**BMI (kg/m^2^)**	≤24 (Ref)				
	>24	0.50(0.20-1.20)	0.120		
**Tumor size (mm)**	≤50 (Ref)				
	>50	2.67(1.25-5.73)	***0.012***	2.16(0.99-4.74)	0.054
**Laterality**	unilateral (Ref)				
	bilateral	2.28(1.35-3.84)	***0.002***	3.99(1.38-11.48)	***0.010***
**Blood flow signal**	No (Ref)				
	Yes	0.97(0.68-1.39)	0.877		
**Surgery type**	BCS (Ref)				
	TM	0.669(0.32-1.40)	0.286		
	VAE	1.69(0.45-6.42)	0.440		

Ref, Reference group. Bold values indicate statistical significance at *p* < 0.05.

### 3.3 Borderline PT

**Figure 4:**
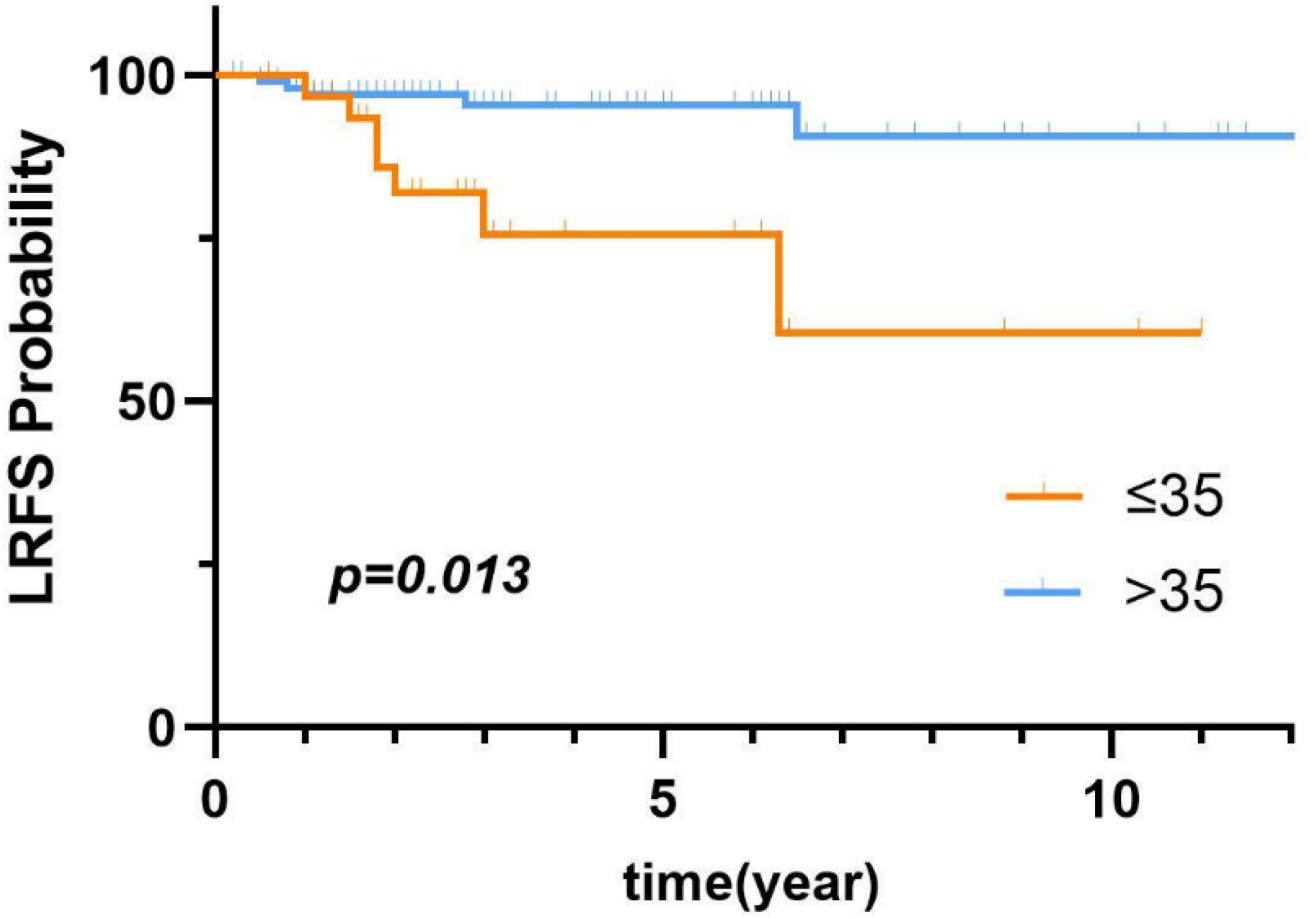
LRFS of borderline PT based on age.

For patients with borderline PT, the median time to LR was 1.9 (IQR, 1.7–6.0) years, the 5-year LRR was 7.7%, the LRR of borderline PT was 9.9%. The median age at initial diagnosis was 47 (IQR, 37–54) years, and the median tumor size was 44.9 (IQR, 28.9–65.0) mm. **Table 4** shows the results of univariate analysis revealed that younger age (≤35 years) was the only factor influencing LR of borderline PT. Compared to those aged ≤35, individuals aged >35 had a significantly lower risk of LR (HR=0.24, 95% CI:0.08-0.68). **Figure 4** shows the comparison of LRFS in borderline PT between patients aged ≤35 and those aged>35.

**Table 4:** Univariate Cox regression models for variables associated with borderline PT.

Characteristics		Hazard ratio (95% CI)	*p*-value
**Age (years)**	≤35 (Ref)		
	>35	0.24(0.08-0.68)	***0.008***
**BMI (kg/m^2^)**	≤24 (Ref)		
	>24	0.51(0.14-1.83)	0.298
**Tumor size (mm)**	≤50 (Ref)		
	>50	1.93(0.67-5.56)	0.226
**Laterality**	Left(Ref)		
	Right	0.76(0.25-2.27)	0.622
**Blood flow signal**	No (Ref)		
	Yes	0.63(0.22-1.81)	0.392
**Surgery type**	BCS (Ref)		
	TM	32.38	0.955
	VAE	0.000	0.948

Ref, Reference group. Bold values indicate statistical significance at *p* < 0.05.

### 3.4 Malignant PT

For patients with malignant PT, the median time to LR was 1 (IQR, 0.5–2.3) year, the 5-year LRR was 13.2%, the distant metastasis rate for malignant PT was 14.2%. The median age at initial diagnosis was 49 (IQR, 36.5–56) years, and the median tumor size was 48.3 (IQR, 32-87.6) mm.

**Figure 5:**
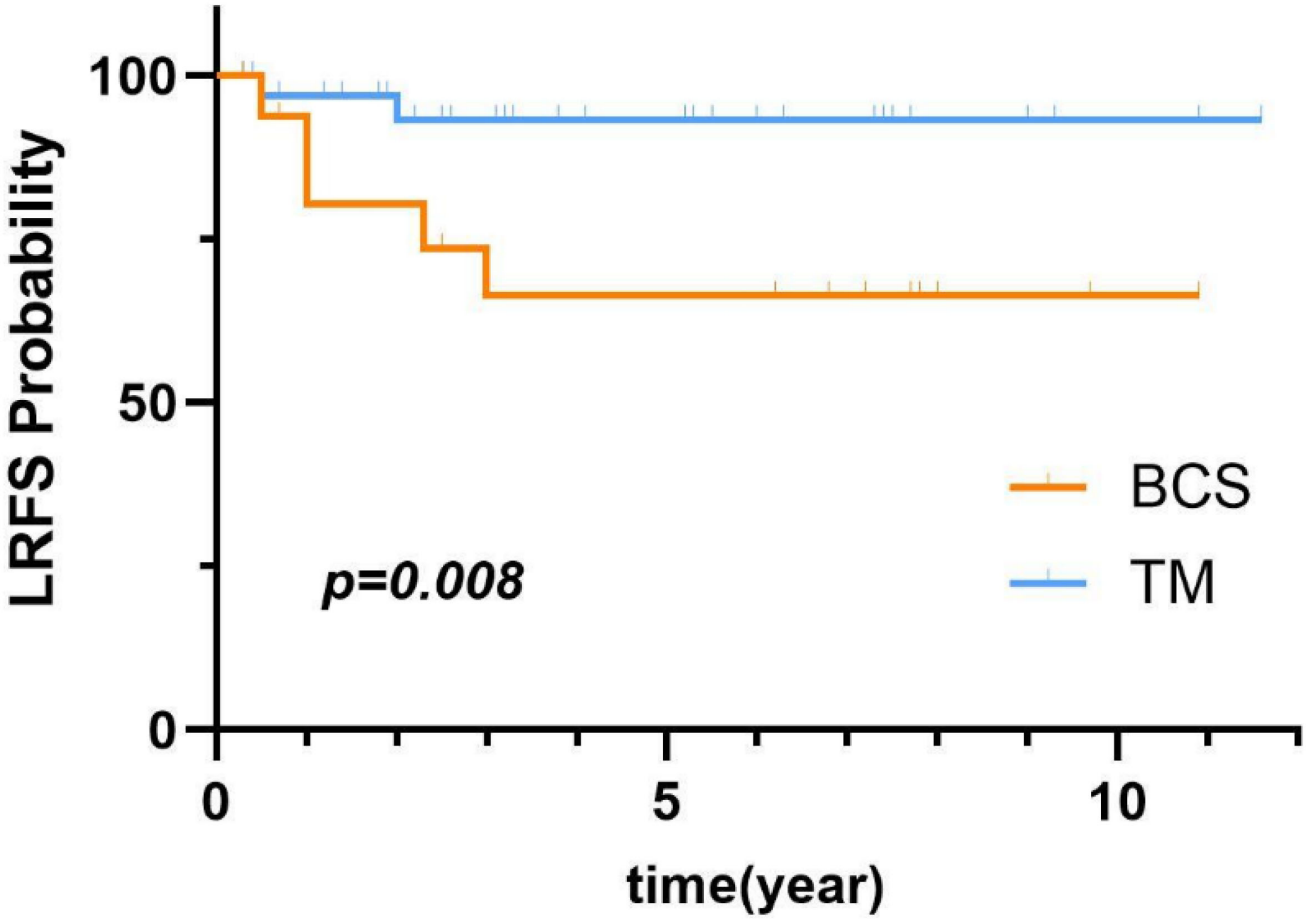
LRFS of malignant PT based on surgery type.

COX univariate analysis showed that the surgery type was a prognostic factor for patients with malignant PT. **Figure 5** illustrates the comparison of LRFS between malignant PT patients who underwent BCS and those who underwent TM. As shown in **Table 5**, univariate analysis revealed that BCS (*p*=0.046) were risk factors for LR of malignant PT. Additionally, compared to BCS, TM significantly reduced the LRR (HR=0.19, 95% CI:0.04-0.99).

**Table 5:** Univariate Cox regression models for variables associated with malignant PT.

Characteristics		Hazard ratio (95% CI)	*p*-value
**Age (years)**	≤35 (Ref)		
	>35	0.51(0.10-2.64)	0.422
**BMI (kg/m^2^)**	≤24 (Ref)		
	>24	0.29(0.04-2.39)	0.248
**Tumor size (mm)**	≤50 (Ref)		
	>50	3.25(0.63-16.88)	0.161
**Laterality**	Left(Ref)		
	Right	0.82(0.18-3.67)	0.796
**Blood flow signal**	No (Ref)		
	Yes	0.81(0.18-3.63)	0.785
**Surgery type**	BCS (Ref)		
	TM	0.19(0.04-0.99)	***0.046***

Ref, Reference group. Bold values indicate statistical significance at *p* < 0.05.

The clinical characteristics of 7 patients with LR of malignant PT are presented in **Table 6**.

**Table 6:** Characteristics of the 7 patients with recurrence of malignant PT.

Age (years)	Recurrence site	Tumor size (mm)	Therapy (before)	Therapy (after)	LRFS	OS
44	Ipsilateral breast	27.0	BCS	TM	1.0	3.5, alive
60	Ipsilateral breast	53	BCS	TM	1.0	2.1, dead
25	Ipsilateral axillary	89.4	TM	ALND	1.8	2.7, alive
40	chest wall	37.5	BCS	adjuvant chemotherapy	0.5	2.3, alive
43	Ipsilateral breast	8.0	BCS	TM	2.5	8.7, alive
55	Ipsilateral breast	28.0	BCS	TM	2.6	8.8, alive
41	chest wall	24.0	TM	surgical resection	0.5	1.0, dead

ALND, axillary lymph node dissection.

## 4 Discussion

This is the retrospective study conducted in China to investigate the clinical and pathological characteristics, as well as the risk factors, for LR of PT in two representative hospitals.

The LRR of benign PT was 5.4%, which is consistent with recent studies reporting between 5% and 10% (12). Among patients with benign PT, 13 (2.1%) were diagnosed with bilateral benign PT, which included 3 (23.1%) with LR. Univariate analysis with the Cox proportional-hazards model identified bilateral PT as a possible risk factor for LR of benign PT. A large retrospective study conducted in 2023 confirmed LR in 7 (24.1%) of 29 patients with bilateral benign PT and suggested that this relatively high LRR might be attributed to genetic (4). In the present study, 71.1% patients with benign PT chose BCS, while only 0.8% chose TM, and 28.1% chose VAE. However, as compared to BCS, patients with tumors < 20 mm in the last 5 years tended to prefer VAE because the cost of ultrasound-guided VAE is approximately 80% less than surgery (13), is less time-consuming, and yields better aesthetics. Most importantly, ultrasound-guided VAE is safe and effective for resection of lesions < 20 mm (14). Therefore, VAE is increasingly preferred for resection of benign tumors.

The LRR of borderline PT was 9.9%. Younger age (≤35 years) was the only factor significantly associated with LR. As compared to younger patients (≤35 years), 5-year LRFS was significantly increased for older patients (>35 years), in agreement with a prior report (15). BSC is most commonly employed for borderline PT. In the present study, there was no significant difference in local control of borderline PT among the three types of surgery.

In this study, 66% of patients selected TM for resection of malignant tumors. Moreover, as a favorable prognostic factor for malignant PT, TM significantly improved LRFS, in agreement with the results of a previous study (16). However, some studies suggest that as compared to TM, BCS did not improve the LRR (17–19). There was no significant difference in oncological outcomes between BCS and TM for early stage malignant PT, thus BCS is preferable (20). Numerous studies have demonstrated an association between TM and reduced risk of LR in patients with borderline and malignant PT. However, no specific surgery type has been found to directly impact cancer-specific survival rates (2).

Large tumor size (>50 mm) is also considered a possible risk factor for LR of malignant PT in previous studies (6, 11, 21, 22). A study conducted in 2013 suggested that small tumor size (<4.0 cm) was associated with increased LRR (23). We attribute these disparate findings to small sample sizes and the possibility of rapid tumor growth within a short period of time, which often occurs with high-grade malignant tumors, indicating more rapid progression of the tumor and a relatively poor prognosis. However, there is currently no reliable data demonstrating a correlation between the tumor growth rate and prognosis (6).

The LRR of malignant PT was 13.2%, with a high metastasis rate of up to 20% (24), indicating a poor prognosis. In this study, 2 (28.6%) of 7 patients with malignant PT experienced LR of the ipsilateral chest wall. One of these patients underwent surgical resection of the chest wall lesion but died of lung metastasis one year later, while the second received chemotherapy. Patients with LR of malignant PT to the chest post-TM reportedly respond well to radiotherapy (22). The National Comprehensive Cancer Network guidelines also recommend adjuvant radiotherapy (25). However, the efficacy of chemotherapy for treatment of malignant PT remains unclear (26). In this study, as most PT patients did not receive radiotherapy or chemotherapy, and due to incomplete treatment records, this study did not include these variables in the analysis. Therefore, large-scale prospective clinical trials are still needed to evaluate the role of adjuvant chemotherapy or radiotherapy in malignant PT.

The relationship between surgical margin status and the risk of local recurrence has garnered significant attention in recent years. Ditsatham et al. reported that surgical margins < 1 cm have no significant impact on local recurrence rates or 5-year disease-free survival in PT patients (27). A recent study revealed that patients with positive surgical margins have over 10 times the risk of local recurrence compared to those with wide margins (>10 mm) and over 6 times the risk compared to those with narrow margins (<10 mm) (28). For benign PT, complete mass resection is sufficient. Multiple studies have demonstrated no correlation between surgical margin status and recurrence risk in benign PT (4), and postoperative adjuvant therapy is unnecessary for these patients. For borderline and malignant PT, narrow surgical margins are linked to a higher risk of local recurrence. Consequently, wide local excision with a surgical margin of ≥1 cm is typically recommended (29). Unfortunately, this study does not address the correlation between surgical margins and recurrence risk due to the lack of accurate margin data from two representative hospitals. Therefore, future studies are encouraged to clearly margin width in order to better elucidate the potential association between surgical margins and the risk of LR, thereby providing stronger evidence to optimize surgical strategies.

In this study, prognosis was better for benign and borderline PT, probably because some patients with small tumors chose observation rather than resection. The lack of pathological evidence for LR has resulted in underestimation of the recurrence rate.

This study has several limitations. Firstly, the median follow-up of 2.8 years is relatively short, longer follow-up is warranted in future studies to further validate our findings. Secondly, the low incidence of the disease resulted in a small sample size, with only 14 cases of borderline PT recurrence and 7 cases of malignant PT recurrence, and 13 (2.1%) were diagnosed with bilateral benign PT, which included 3 (23.1%) with LR introducing potential statistical bias. Unfortunately, among the 7 patients with recurrent malignant PT, some were unable to provide detailed information about their recurrence during follow-up, limiting the ability to perform a comprehensive comparison before and after recurrence. Thirdly, descriptions and investigations of certain pathological features of PT are lacking. In future research, we plan to expand the sample size and incorporate additional pathological features to provide more comprehensive and compelling results.

These findings, particularly the identification of LR risk factors stratified by tumor grade, may contribute to more personalized risk assessment in clinical practice. Future studies could explore how these factors might be incorporated into decision-making algorithms for patients with PT and other soft tissue tumors.

## 5 Conclusion

The results of this retrospective dual-center study found that bilaterality was a risk factor for LR of benign PT, younger age (≤35 years) is an unfavorable prognostic factor for borderline PT, and BCS is a risk factor for LR of malignant PT.

## Data Availability

The original contributions presented in the study are included in the article/supplementary material. Further inquiries can be directed to the corresponding authors.
